# Hospital-Based Medical-Legal Partnerships for Complex Care Patients: Intersectionality and Ethics Considerations

**DOI:** 10.1017/jme.2023.154

**Published:** 2023

**Authors:** Megha Garg, Jennifer Oliva, Alice Lu, Marlene Martin, Sarah Hooper

**Affiliations:** 1: THE UNIVERSITY OF CALIFORNIA SCHOOL OF MEDICINE, SAN FRANCISCO, CA, USA; 2: SAN FRANCISCO VETERANS AFFAIRS MEDICAL CENTER, SAN FRANCISCO, CA, USA; 3: INDIANA UNIVERSITY, BLOOMINGTON, IN, USA; 4: THE UNIVERSITY OF CALIFORNIA COLLEGE OF LAW, SAN FRANCISCO, CA, USA

**Keywords:** Medical-Legal Partnerships, Guardianship, Intersectionality, Complex Care Patients, Health Disparities

## Abstract

Health systems are integrating medical-legal partnerships (MLPs) into clinical care and increasingly center “complex care” patients. These patients have intersecting medical and social needs and often face systemic inequities that exacerbate their chronic health conditions. This paper describes a role for MLPs in hospital quality initiatives; examines the ethics of MLPs assisting with guardianship and institutionalization of hospital patients including marginalized groups; and advocates for MLP interventions designed to address intersectional and ethical concerns.

American health systems have invested over $2.5 billion to address social determinants of health in recent years.^1^ This includes the increased adoption of medical-legal partnerships (MLPs), which integrate lawyers’ expertise into the health care setting as a patient care intervention to improve health outcomes, reduce costs, and satisfy regulatory requirements. MLPs increasingly center “complex care” patients who have intersecting medical and social needs and are often among the most marginalized members of a community. They frequently experience a combination of functional impairment (e.g., physical or cognitive), chronic illness, mental health disorders, substance use disorders, poverty, low educational attainment, housing instability, and justice system involvement.^2^

The complex care population disproportionately includes older people, women, and people with disabilities, all groups for whom legal services are an essential need.^3^ People with complex care needs often face significant systemic inequities, such as racism, ableism, misogyny, and ageism, that exacerbate their chronic health conditions. Structural racism compounds the medical and social risks faced by minoritized and racialized groups.^4^ In addition, people with complex care needs can pose high financial risks to health systems or payors, which may incentivize biased or inappropriate care decisions.^5^ Therefore, medical, legal, and equity focused perspectives are essential in caring for people with complex care needs.

We examine the role of hospital-based MLPs as an intervention for patients with complex care needs and explore the model’s benefits and potential risks vis-a-vis marginalized patients.

Older adults are disproportionately likely to have complex medical needs.^6^ They encounter significant barriers to care and care coordination and experience worse health outcomes than younger adults.^7^ They also are more likely to be admitted to a nursing home than receive in-home or community-based care, which reflects Medicaid’s structural bias toward nursing home care and the program’s lack of adequate infrastructure to support community living.^8^

MLPs can help address the factors that lead to preventable healthcare utilization and serve as integral components of the hospital team, particularly in care transitions and discharge planning, which can benefit both patients and the health system. Legal interventions in this context, however, raise ethical and civil rights questions that must be addressed thoughtfully to avoid unintended harm to complex care populations.

The limited literature on MLP ethical considerations focuses on end of life, pediatric, and adolescent care contexts. Issues include the preservation of independent professional judgment, navigation of confidentiality and attorney-client privilege, and the complexities that attend to representing a client’s stated best interests when the client has diminished capacity or their decisions are at odds with the medical team’s recommendations.^9^ This paper discusses these issues within the context of hospitalization and discharge decisions and adds ethical considerations for legal and medical teams, as well as health systems more broadly.

We examine the role of hospital-based MLPs as an intervention for patients with complex care needs and explore the model’s benefits and potential risks vis-a-vis marginalized patients. The paper begins by describing the emerging role for MLPs with the complex care patient population, including MLPs’ impacts on quality initiatives to improve hospital patient flow and discharge planning. It then details the ethical tensions and risks presented by MLP guardianships and institutional placements to hospitalized patients, with an emphasis on patients who identify as racial minorities, women, and people with disabilities. The paper concludes by proposing steps to mitigate these risks and advocating for the design and implementation of MLPs through rigorous intersectional, critical, and ethical lenses.

## I. Role of MLPs in Hospital-Based Complex Care Initiatives

Patients with complex care needs often experience prolonged hospitalizations for non-acute reasons.^10^ While numerous factors contribute to this dynamic, this frequently occurs because the patient lacks a safe or affordable placement option outside of the hospital.^11^ Patients rarely prefer prolonged hospitalization, which is associated with serious health risks, including hospital-acquired complications, delirium, social isolation, depression, physical deconditioning, and loss of autonomy (e.g., hospital rules prohibiting patients from walking outside of the facility without permission). For healthcare workers, prolonged unnecessary patient hospitalizations contribute to clinician burnout.^12^ Prolonged hospitalization also incurs significant costs to the health care system, including the direct costs of individual patient hospitalization as well as the downstream effects of hospital bed nonavailability.^13^ Placement problems often are related to questions about patient capacity, legally-recognized decisionmakers, unsafe or unavailable housing, financial resources, and insurance coverage, all of which are best addressed with legal assistance.

MLPs provide benefits for patients in areas that impact hospitalization, including improved access to housing, access to services, and reduced psychosocial distress.^14^ Studies demonstrate an association between addressing legal barriers to discharge and decreased length of stay for patients with complex care needs.^15^ Consequently, there is an emerging role for MLPs in quality initiatives that aim to improve hospital discharge planning and mitigate placement challenges.

The University of California, San Francisco-University of California College of Law, San Francisco Medical Legal Partnership for Seniors (MLP for Seniors) at the San Francisco Veterans Affairs Medical Center is one example of a quality initiative focused on older (over age 60) patients with complex care needs. In the MLP for Seniors, attorneys with elder law expertise help patients navigate access to housing, long-term care, and benefits as well as assist patients with capacity concerns and Medicaid eligibility. MLP for Seniors contributed to an approximately $800,000 cost savings to the health system by reducing the length of hospitalization for just five patients.^16^ As part of an interdisciplinary care team, MLP for Seniors helped realize an almost 40 percent reduction in patients admitted to the hospital for over 30 days over a six-month period.^17^

Hospitalization can serve as an entry point to the health care delivery system for patients with legal needs who might not otherwise have sought legal services in the community, and legal partners add value to the care patients receive.^18^ When a health care clinician identifies a patient who may be at risk for a prolonged hospitalization due to lack of in-home care support or financial resources, legal partners can assist with upstream factors, including advance medical and financial planning, to prevent unnecessary utilization of health care resources and increase patient satisfaction.^19^ Robust MLPs can coordinate anticipatory interventions at seminal moments, such as at the time of a new dementia diagnosis, to prevent the need for acute legal intervention such as guardianship.^20^

MLPs also contribute to the collective legal education of the multidisciplinary health care workforce, including clinicians and social workers, as well as assist in the development of patient education materials required by Medicare quality metrics, some of which are particularly relevant to older, complex care populations.^21^ By addressing these myriad factors, MLPs can serve patient needs, assist clinicians and social workers with knowledge gaps, and potentially save the system money by reducing prolonged or unnecessary stays. Many MLPs receive funding directly from the health systems they serve due to their shared savings potential and the benefits they extend to multiple stakeholders.^22^

## II. Ethical and Intersectionality Considerations for MLPs in Hospital-Based Complex Care Initiatives

Health systems’ funding of programs that address patient legal and social needs, like MLPs, can be driven by a desire to improve health and health equity and by an expectation of return on investment in the form of cost savings for decreased length of inpatient stays. Singular focus on financial gain or cost metrics, however, may result in unintended consequences for patient care because heightened emphasis on cost savings risks incentivizing biased or inappropriate care decisions. Narrow attention to cost benchmarks also can provoke decisions to close beneficial programs that fail to achieve profit objectives. These pressures jeopardize the longstanding aim of MLPs as interventions that prioritize patient goals and maximize patient civil rights (e.g., by asserting rights to health care decision making or disability accommodations) and should be strongly resisted. If viewed exclusively as a vehicle to help the health system reduce utilization and save money, MLPs may be disincentivized to pursue legal actions that are at odds with cost-reduction goals, even when they are in the patients’ best interests.

An example is when hospital teams seek either guardianship or institutional placement for patients under the theory that such action will expedite discharge or prevent future health care utilization.^23^ Hospital teams often believe that such pursuits are either the only options, or the most ethical courses of action to take in the patients’ interests, and MLP lawyers may sometimes agree. MLPs, however, must be cautious when assisting in guardianships or institutional placements because such interventions infringe on patients’ rights. Guardianship proceedings are, by their nature, intended to impair a patient’s civil rights because they remove an individual’s right to make certain decisions for themselves, such as where they will live, what care they receive, and how they arrange their finances. In addition, the Americans with Disabilities Act makes clear that institutional placements that fail to maximize community options constitute a form of segregation and discrimination that violates federal law.^24^ MLPs must evaluate their professional ethical obligations as well as patient equity factors before taking such actions, and should ensure that health care teams embed such considerations in the decision-making process as a matter of hospital practice and policy. Such ethical obligations become particularly relevant when the MLP is funded by the health care partner and the desire to reduce hospital utilization motivates the partnership.

### Professional Ethics

Patients with complex care needs, generally, and those with dementia or who lack decision-making capacity, specifically, are predisposed to prolonged hospitalizations due to the challenges that attend to arranging a safe discharge plan.^25^ Physicians may be capable of identifying safety concerns for patients living in the community without adequate supports, but they often lack knowledge regarding capacity assessment or placement options, as well as the implications of pursuing guardianship and legal placement.^26^ In the inpatient setting, physicians often consider legal guardianship as an option when arranging for the safe discharge of individuals without identified family or healthcare decision makers. During hospitalization, health care teams may predominantly focus on optimizing the guardianship process rather than evaluating the initial decision to pursue guardianship, and MLP lawyers may be asked to help expedite the guardianship process.^27^

While MLP lawyers work collaboratively with health care teams to reach patient goals, they are ethically obligated to represent patients and not hospitals.^28^ The pursuit of an involuntary guardianship or institutional placement constitutes a *per se* conflict with the patient’s rights and interests that the patient‘s own attorney generally cannot undertake.^29^ However, professional ethics rules in some states open the door for lawyers to seek protective action over their own clients in limited situations.^30^ Despite the potentially significant incentives for MLP lawyers to pursue such actions, they can and should be constrained by other ethical considerations.

Clinicians must ethically balance the risks and benefits of all patient interventions.^31^ This includes the need to consider the harms that can result from social interventions like guardianship, even when those interventions are well-intended. The pursuit of guardianship for patients can cause moral distress for physicians as they attempt to balance a patient’s safety and autonomy. Unfortunately, clinicians often underestimate patient capacity and overestimate the benefits of guardianship. Health care teams must perform due diligence and fully explore alternatives to guardianship, and MLPs can provide guidance and education to support those efforts. Furthermore, when a caregiver or surrogate decision maker is involved in the patient’s care, it is important for clinicians to assess whether those individuals are making decisions in the patient’s best interests. MLPs can serve as important resources in exploring less restrictive options before determining that guardianship is the appropriate strategy for achieving a patient’s safe discharge goals.^32^ For example, the MLP for Seniors was successful in improving guardianship assessment processes by standardizing capacity assessment procedures via geriatrics and legal expertise; creating a checklist and analytical framework for alternatives to guardianship, such as appointment of surrogates or representative payees; and advocating for benefits like funding for caregivers or home services that support community living. The MLP for Seniors team also facilitated meetings between hospital staff and community agencies, such as the public guardian’s office, to increase mutual understanding of the complex community-level challenges that these stakeholders were collectively facing and the limits of hospitalization and guardianship to address them.^33^

Regardless of the applicable state’s professional ethics rules, MLPs and health systems that place patients under guardianship or institutionalize patients while failing to explore alternatives that may be equally or more beneficial to patients are contrary to the MLP model. The MLP model ultimately aims to advance health justice — addressing the oppressive societal structures, including racism, poverty, and discrimination, that lead to health inequity.^34^

### Least Restrictive Alternatives

Thirty-nine states’ statutes specifically mandate that guardianship be granted only where it is the “least restrictive” means to address an incapacitated person’s needs. With or without this statutory requirement, a core obligation of the MLP should be to critically evaluate whether guardianship or institutional care is necessary. The first consideration is whether other patient supports are available to avoid the need for these interventions. If they either are not available, or have not been successful, the team must query “why”? For example, was an individual characterized as “failing in the community” simply because they lack the ability to pay for a part-time caregiver who may have allowed them to continue living independently? An attempt to identify resources to augment caregiving options is essential in such a case prior to proceeding with guardianship or institutional care. A second consideration is whether rights-stripping interventions will resolve a problem that other supports have been unsuccessful at mediating. Guardianship arrangements are neither guaranteed to provide more safe placement options for patients in which there is a nursing home bed shortage, nor create new financial resources to finance caregiving. Throughout the process, the MLP team must carefully weigh the benefits and risks of guardianship and institutional care against alternative options. In weighing the risks and benefits, MLPs must include a third consideration affecting the marginalized populations they serve: implicit bias and equity.

### Implicit Bias and Equity Considerations

Guardianship is more often pursued for the most marginalized patients who lack meaningful financial resources and family advocates. Studies from New York and Florida indicate that women and racialized and minoritized individuals are overrepresented in the population under guardianship, which reflects existing structural gender and racial inequities and suggests that implicit bias may also play a role.^35^ The communities at highest risk for guardianship and institutional placement proceedings are those that have experienced generational and lifetime discrimination, which is often why they are sicker and disempowered.^36^

The MLP model is designed to address the social determinants of health of patients with complex care needs, including factors like structural racism, ableism, and ageism, and barriers to access to justice. MLPs have an opportunity to adopt anti-racist missions and address their own role in facilitating or deploying structurally racist policies. As health systems build programs like MLPs to address the social determinants of health, it is critical that MLPs capitalize on the opportunity to explicitly address structural racism in their mission and to embed antiracism into institutional practices.

Despite the common understanding among health care teams that guardianships and institutional placements are health- and patient-safety focused interventions, those legal processes invoke the carceral system, which is particularly problematic for historically oppressed communities. Liat Ben-Moshe argues that the penal system targets people with disabilities and that diverse sites of confinement (including hospitals) have emerged as a result.^37^ Scholars have described psychiatric hospitalization as imprisonment of the innocent without trial,^38^ and have advanced arguments to entirely abolish involuntary commitment.^39^

MLPs must consider the impacts of these interventions on the patients they aim to serve and embed opportunities for reflection and interruption of bias in assessment processes. For instance, MLPs should examine the ways in which a patient’s identity and personal history may be informing professional judgments about their decision-making capacity and community living options. Teams could instate “blinded” case consults in which age, gender, and race are removed and the team focuses on functional strengths, needs, and the resources needed and desired. In weighing benefits and risks of involuntary interventions, health care teams and lawyers should consider the patient’s subjective experience of trauma, loss of control, damage to the patient-physician relationship, and loss of trust in the health care system as key risks that may need to be more heavily weighted as risks in historically marginalized patient populations.^40^

## III. The Path Forward

Though the potential harms of hospital-based MLPs for patients with complex care needs are apparent, their benefits outweigh their risks, so long as the partnership structures and designs its interventions through rigorous intersectional and ethical lenses. To be successful, MLPs must consider potential for implicit bias and undue influences, provider training, program evaluation, and deliberate anti-racist practices.

MLPs must identify the biases and financial motives that may influence MLPs for patients with complex care needs and acknowledge that even well-intentioned interventions can exacerbate existing structural inequities and erode trust in health systems. While it is reasonable for health systems to anticipate and strive for a return on investment from MLP programs, the fundamental motivation for an MLP cannot be anything other than its service obligations to its patients.

MLP legal staff represent the patient. They do not serve as legal counsel for the hospital. It, therefore, is incumbent upon the MLP lawyer to avoid anchoring only on the medical team’s legal question when developing patient recommendations. Instead, MLP attorneys should holistically approach the individual patient’s context and examine any biases, implicit or explicit, that may be contributing to the situation.

MLP evaluation must constitute a key component of the funding plan and structure of the enterprise. Data should appropriately capture MLP process and outcome measures such as the savings, metrics impacts, costs, and benefits to all stakeholders, including patients, clinicians, and the multidisciplinary care team. Patient demographic data also should be collected to assess racial, gender, age, socioeconomic, and disability disparities or bias in hospital and MLP interventions. In addition, MLP evaluation should include less tangible factors, such as the model’s impact on mitigating physician burnout and attrition of staff when appropriate supports for patients and care teams are in place in a health system. While these factors can be extrapolated to contribute to the financial bottom line, the mission of the organization to serve patients must remain paramount.

MLPs should incorporate training for both health care and legal teams in the ethical, legal, and civil rights issues inherent in interventions such as guardianship and institutionalization, with specific content training on alternatives to guardianship and placement options in the community. At the San Francisco VA Medical Center, the training provided by MLP for Seniors lawyers to the health care teams led to a complete process overhaul of how guardianship was pursued. MLP assessment and guidance of health care teams led to inpatient standardization of patient capacity evaluations by geriatrics physicians and increased direct communication with the local public guardian’s office. When the health care teams learned that guardianship would not always resolve issues of finances or institutional bed availability, they often abandoned the pursuit of guardianship altogether in favor of alternatives that prioritized patient independence in the community.

Clinical workflows should be built with MLP guidance that operationalize less restrictive options for patients. To the extent health systems and lawyers advocate for policy change, the focus of their efforts should not be on expediting guardianship and institutional placement processes, but on addressing the underlying social and community risk factors that give rise to their perceived need. Examples include the expansion of Medicaid home and community-based services and enhanced availability of supportive housing, pro bono fiduciary services, and access to free legal help for person-centered care planning.

The MLP model is designed to address the social determinants of health of patients with complex care needs, including factors like structural racism, ableism, and ageism, and barriers to access to justice. MLPs have an opportunity to adopt anti-racist missions and address their own role in facilitating or deploying structurally racist policies.^41^ As health systems build programs like MLPs to address the social determinants of health, it is critical that MLPs capitalize on the opportunity to explicitly address structural racism in their mission and to embed antiracism into institutional practices. Examples include exploring the history and politics of the communities served by the MLP; examining the privilege of the health care and MLP providers; teaching patients self-advocacy skills such as how to document encounters of racial discrimination; and getting feedback from community members on their approaches to case management. A health justice-oriented vision for MLPs would allow such partnerships to evaluate their role in perpetuating or legitimizing oppressive societal structures, and contemplate how they might contribute to broader racial and community justice goals.^42^

## Conclusion

MLPs are increasingly adopted in hospital-based settings for patients with complex care needs. Consequently, it is important for MLPs to look beyond the benefits that the partnership inures to the health system and understand patient risks. Ethical and intersectionality considerations are crucial with regard to interventions that involve guardianships and institutional placements, especially for marginalized populations. MLPs must take care to prevent patient harm and perpetuation of structures of oppression through their work. MLPs can improve individual patient and population health when they are intentionally designed to evaluate for bias, address structural racism, and advocate for the least restrictive options to serve a patient’s needs.
